# Control of Macromolecule Chains Structure in a Nanofiber

**DOI:** 10.3390/polym12102305

**Published:** 2020-10-08

**Authors:** Dan Tian, Ji-Huan He

**Affiliations:** 1 School of Science, Xi’an University of Architecture and Technology, Xi’an 710049, China; tiandan@xauat.edu.cn; 2 National Engineering Laboratory for Modern Silk, College of Textile and Clothing Engineering, Soochow University, 199 Ren-Ai Road, Suzhou 215123, China

**Keywords:** air vortex, electrospinning, macromolecule chain, nanofibers, DNA-like structure

## Abstract

Mechanical property is one of the most important properties of nanofiber membranes. Electrospinning is widely used in the preparation of nanofibers due to its advantages such as good stability and easy operation. Compared with some nature silk, the mechanical properties of nanofibers prepared by electrospinning are poor. Based on the principle of vortex spinning and DNA structure, this paper designed an air vortex electrospinning device that can control the structure of macromolecular chains in nanofibers. When a weak air vortex is generated in the electrospinning process, the macromolecule chains will entangle with each other and form a DNA-like structure so as to improve the mechanical property. In addition, when a strong air vortex is generated during the electrospinning process, the nanofibers will adhere to each other, thereby enhancing the mechanical property and enlarging the pore size.

## 1. Introduction

Vortex spinning is a pneumatic spinning method and widely used in the textile industry [1,2,3]. It can be viewed as a refinement of jet spinning, or a natural development in fascinated yarn technology, which enables the yarns to exhibit better evenness and tenacity values [4,5]. As we know, DNA has a twisted double helix structure with replication property [6,7]. Similarly, in the yarn spinning, twisting is also one of the most important steps, which influences the geometry of spinning triangle and effects the properties of spun yarns directly. Regarded as the most straight and simplest method to fabricate nanofibers [8,9,10], the electrospinning is used to prepare various materials for different applications [11,12,13]. On the other hand, as a key factor, the mechanical property will determine the application range of nanofiber membranes [14,15,16]. Therefore, how to further improve the mechanical property of nanofiber membrane has always been an important problem for researchers. We tried to control the macromolecule orientation in a nanofiber before, but its mechanical property was very poor [17]. In this paper, inspired by DNA structure and based on vortex spinning, we used the air vortex to control macromolecule chains structure in a nanofiber, and designed an air vortex electrospinning device for fabricating nanofibers whose macromolecule chains have a DNA-like structure. We also studied and discussed the effect of the air vortex strength on macromolecule chains. This paper aimed at demonstrating that the air vortex has an important effect on macromolecule chains in a nanofiber and between nanofibers in the electrospinning process. So as to improve the mechanical property of nanofiber membranes.

## 2. Experimental

### 2.1. Materials

Polyvinyl alcohol (PVA) 1799 with an average molecular weight of 200,000 g/mol was purchased from Shanghai Aladdin Biochemical Technology Co., Ltd. (Shanghai, China), and used as received, alcoholysis degree of the PVA was 97.5–99.0 mol%. Polyacrylonitrile (PAN) with an average molecular weight of 150,000 g/mol was purchased from Beijing Lark Branch Co., Ltd. (Beijing, China) and used as received. N, N-dimethylformamide (DMF) was purchased from Shanghai Chemical Reagent Co. Ltd. (Shanghai, China), and used as received.

### 2.2. Instrumentation

Electrospun nanofiber’s morphology was analyzed using an S4800 cold field scanning electron microscope (SEM, Hitachi S-4800, Tokyo, Japan). To determine the diameter distribution of nanofibers, we randomly selected 500 nanofibers from 50 SEM images, and measured them with ImageJ software (National Institute of Mental Health, Bethesda, MD, USA), finally calculated the average value. Mechanical property was measured by the material testing machine (INSTRON-3365, INSTRON Company, Norwood, MA, USA). The pore size distribution was measured for the nanofiber membranes by a capillary flow porometry (Porometer 3G, Quan-tachrome Instruments, Boynton Beach, FL, USA). FTIR spectra of nanofiber membranes were obtained on a Fourier transform infrared (FTIR) spectroscopy (Nicolet5700, Thermo Nicolet Company, Waltham, MA, USA).

### 2.3. Solution Preparation

1.6 g PVA powders were dissolved in 18.4 g deionized water, then the mixtures were magnetically stirred on the heating magnetic stirrer (DF-101S, Xinrui Instrument Inc., Beijing, China) at the temperature of 80 °C for 3 h to achieve homogeneous solution. 1.6 g PAN were dissolved in 18.4 g DMF, then the mixtures were magnetically stirred on the heating magnetic stirrer (SL-5200DT, Nanjing Shunliu Instrument Co. Ltd., Nanjing, China) for 4 h to achieve homogeneous solution.

### 2.4. Electrospinning Process

The obtained solution was put into a 10 mL syringe, which was mounted in a syringe pump (JZB-1800, JYM Instrument Inc., Changsha, China). A high voltage power supply (DW-P303-1ACF0, High Voltage Electronics Co., Tianjin, China) was used in our experiment. The voltage was 18 kV, the flow ratio was 1 mL/h, the distance between the needle and the collector was 20 cm, the temperature was 24 °C, and the relative humidity was 37%. We repeated the experiment approximately 15–20 times.

**Figure 1:**
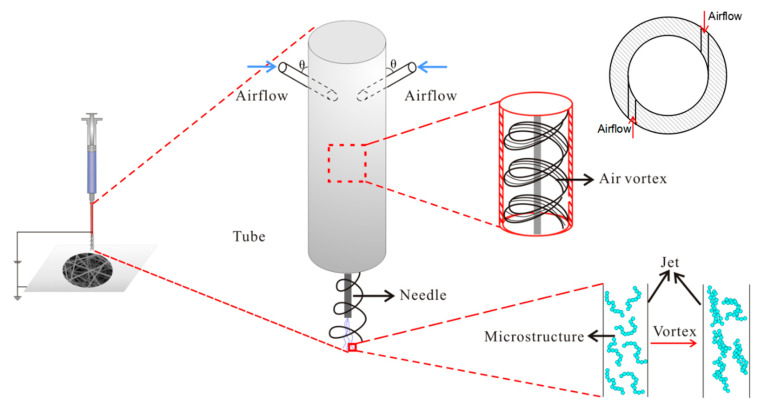
Air vortex electrospinning device.

Based on the working principle of air-jet vortex spinning, in order to improve the mechanical properties of nanofiber membranes, we used vortex in the electrospinning process for the first time and designed an air vortex electrospinning device. Figure 1 shows the experimental device and schematic diagram, as well as a cross-sectional view of the air inlet. The air flow was pumped in a direction almost tangent to the tube wall, and the angle θ with the needle is 90°. As shown in Figure 1, the device differs from the general electrospinning device is that a tube is surrounded by the needle. In this experiment, the length of the needle is 26 mm, which is located in the center of the tube, and the lower edge of the tube is 5 mm higher than the bottom end of the needle. The needle is exposed. The upper end of the tube is closed, the lower end is open, and the upper end is tightly connected with the periphery of the syringe, and the uppermost end of the tube is higher than the uppermost end of the needle. The tube is 40 mm long and 5 mm in diameter. At a distance of 5 mm from the upper end of the tube, have two circular holes with a diameter of 2 mm. The compressed air was pumped into the tube via the two holes. After the air flow was pumped into the tube, it constantly collides with the tube wall in the form of turbulence, and finally formed an air vortex in the tube, like the vortex spinning [18,19,20,21]. Afterwards, the air vortex continues to flow downwards, and had an effect on the jet in the electrospinning process and caused the macromolecular chains in the jet to be entangled. Similar to vortex spinning, the nanofibers were twisted into yarn under the action of vortex. As shown in Figure 1, in this experiment, the macromolecular chains originally dispersed inside the jet were entangled with each other under the action of the vortex. Finally, these jets were stretched and refined under the action of the electric field force to form nanofibers and then deposit on the collector. At this time, the macromolecular chains inside the prepared nanofibers would be entangled with each other. During the electrospinning process, the speed of the air flow was kept constant, so the strength of the air vortex was also constant.

## 3. Results and Discussion

### 3.1. Air Vortex Electrospinning

In this part, we discussed the influence of air vortex on nanofibers. PVA and PAN nanofibers were prepared using the air vortex electrospinning device. The electrospinng condition were all the same. The voltage was 18 kV, the flow ratio was 1 mL/h, the distance between the needle and the collector was 20 cm, the temperature was 24 °C, and the relative humidity was 37%.

#### 3.1.1. Morphological Characterization (SEM)

**Figure 2:**
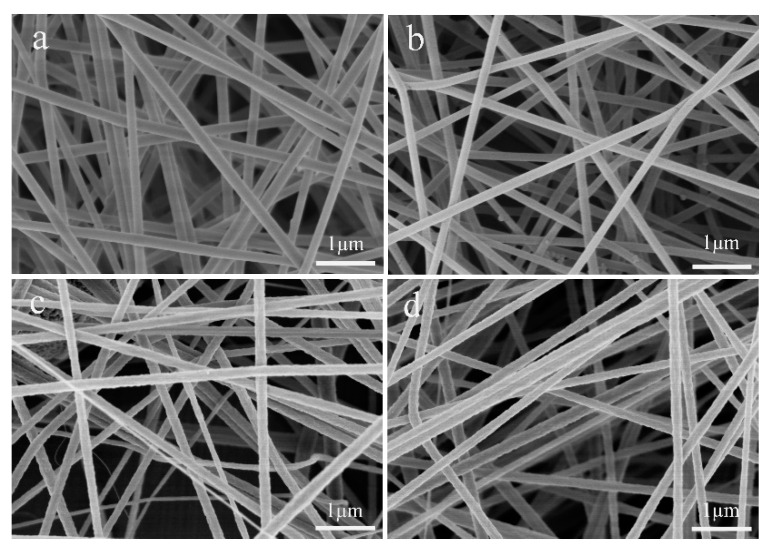
SEM images of nanofibers prepared by different electrospinning device. (**a**) PVA nanofibers prepared by traditional electrospinning device; (**b**) PVA nanofibers prepared by air vortex electrospinning device; (**c**) PAN nanofibers prepared by traditional electrospinning device; (**d**) PAN nanofibers prepared by air vortex electrospinning device.

Figure 2 shows the SEM images of nanofibers prepared using different electrospinning devices and different polymer solutions. Table 1 shows the average diameter of these nanofibers. It can be seen from Figure 2 that the PVA nanofibers and PAN nanofibers prepared by different electrospinning devices have no significant changes in morphology. This reflects that the air vortex only has an effect on the internal structure of the nanofibers. Through the measurement of the average diameter, we known that the nanofibers prepared by the air vortex electrospinning device have a finer diameter. This is because under the action of the air vortex, the vortex exerts a weak stretching force on the jet during spinning. In addition, since the macromolecular chains in the nanofibers are entangled with each other, their position distribution in nanofibers are more concentrated. Therefore, the space they occupied will be relatively small, and the diameter of the nanofiber will be smaller. Besides, the internal macromolecular chains of a certain concentration of spinnable polymer solution are entangled with each other [22,23,24]. So, the high shear stress and elongation stress caused by the vortex flow may reduce the viscosity of the spinning solution, thereby reducing the diameter of the prepared nanofibers.

#### 3.1.2. Mechanical Property

**Figure 3:**
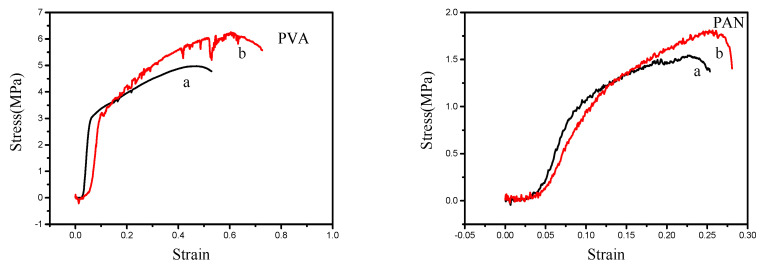
Mechanical property tests for PVA and PAN nanofiber membranes. (**a**) Nanofiber membranes prepared by traditional electrospinning device; (**b**) Nanofiber membranes prepared by air vortex electrospinning device.

The entanglement of macromolecular chains inside nanofibers will greatly affect the mechanical properties of nanofiber membranes. Figure 3 shows the mechanical property test curves of PVA and PAN nanofiber membranes prepared by different electrospinning devices. It can be seen that for the two different polymers, the breaking strength and breaking elongation of the nanofiber membranes prepared by the air vortex electrospinning device are larger than those prepared by the traditional electrospinning device. This is because when the air vortex act on the spinning jet, the macromolecular chains in the jet are entangled with each other, and the macromolecular chains in the prepared nanofibers will also be entangled. So, when the tensile force acts on the nanofiber membranes, the friction force that needs to be overcome due to the entanglement of the macromolecular chains inside the nanofiber will greatly increase, resulting in an increase in the maximum breaking strength and the toughness of the nanofiber membranes. From the SEM image, we can see that the diameter of the nanofibers prepared by the air vortex electrospinning device has some reduced, but there is no adhesion between the nanofibers, the morphology of the nanofibers has little change. In this case, the mechanical properties of the nanofiber membranes have been improved. It is further proved that it is the results of the entanglement of macromolecular chains inside the nanofibers.

#### 3.1.3. Fourier Transform Infrared (FTIR) Spectroscopy

**Figure 4:**
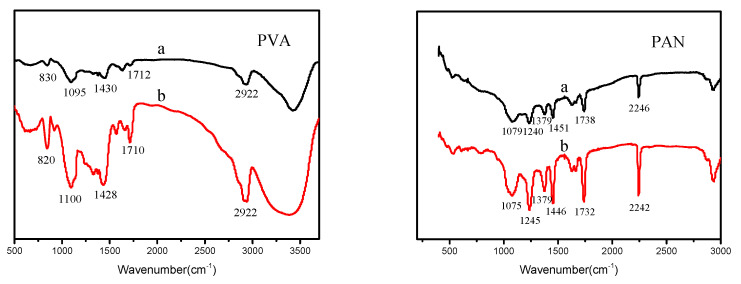
FTIR spectra of PVA and PAN. (**a**) Nanofiber membrane prepared by traditional electrospinning device; (**b**) Nanofiber membrane prepared by air vortex electrospinning device.

Figure 4 present the FTIR spectra of PVA and PAN nanofibers. The infrared spectrum of PVA, the bands at 820 cm^−1^ attribute to C-C vibration, 1100 cm^−1^ relate to C-O stretching vibration, C-OH vibration at 1410 cm^−1^, 1710 cm^−1^ attribute to C=O stretching vibration, CH_2_ bending and stretching vibration at 2922 cm^−1^ [25,26]. The infrared spectrum of PAN, the adsorption peaks of stretching vibrations at 2242 cm^−1^ (C≡N), 1732 cm^−1^ (C=O of ester group), 1446 cm^−1^ (C-H bending in CH_2_) and 1245 cm^−1^ (C-N bending) are clearly observed [27,28,29]. From the spectra, since no new band as well as any shift at absorbance bands is observed in nanofibers, it can be concluded that no chemical bonds have been created. However, in the infrared spectrum of nanofiber membranes fabricated by air vortex electrospinning device, some peaks were strengthened, this may be due to the entanglement of macromolecule chains, which enhancing the vibration.

#### 3.1.4. Gas Permeability

**Figure 5:**
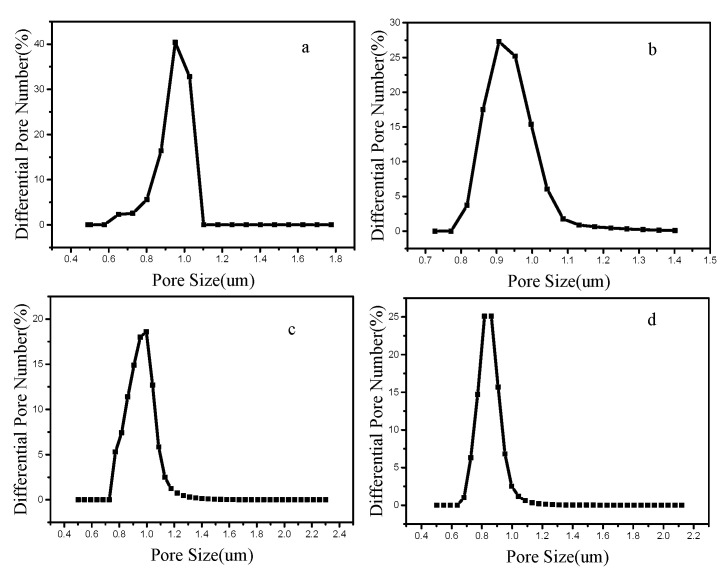
Pore size distributions of the nanofiber membranes. (**a**) PVA nanofiber membranes prepared by traditional electrospinning device; (**b**) PVA nanofiber membranes prepared by air vortex electrospinning device (1 m/s); (**c**) PAN nanofiber membranes prepared by traditional electrospinning device; (**d**) PAN nanofiber membranes prepared by air vortex electrospinning device.

The pore size distributions of PVA and PAN nonofiber membranes were measured by a capillary flow porometry. Figure 5 and Table 2 illustrate the pore size distributions of these nonofiber membranes prepared by traditional electrospinning device and air vortex electrospinning device.

It can be seen from Figure 5 and Table 2 that the pore size of the nanofiber membrane prepared by the air vortex electrospinning device is smaller. This is because the addition of air vortex makes the distribution of nanofibers denser. At the same time, the entanglement of the macromolecular chains inside the nanofibers makes the nanofibers thinner, and the pore size of the nanofiber membrane is positively correlated with the diameter of the nanofibers [30,31,32]. When the nanofiber diameter is smaller, the pore size of the nanofiber membrane is correspondingly smaller. However, because the diameter does not change much, the change of the pore size is also small.

### 3.2. Different Air Vortex Strength

In the previous section, we used the air vortex electrospinning device and selected two different polymer solutions for spinning and discussed the influence of the air vortex on the macromolecular chains inside the nanofibers. The previous experiments found that the weak air vortex has a greater impact on the macromolecular chains inside the nanofibers but has almost no effect on the nanofibers. In addition, the use of the air vortex improves the mechanical properties of the nanofiber membranes.

In this section, we changed the intensity of the air vortex by selecting different airflow speeds and discussed the influence of air vortex strengths on the macromolecular chains inside the nanofibers and the interaction between nanofibers. In this section, 8% PVA solution was used for spinning, and the speed of the pumped airflow was selected as 1 m/s, 2 m/s, 3 m/s, 4 m/s and 4.5 m/s, respectively. The silk conditions and spinning environment were also consistent with those described above.

#### 3.2.1. Morphological Characterization

**Figure 6:**
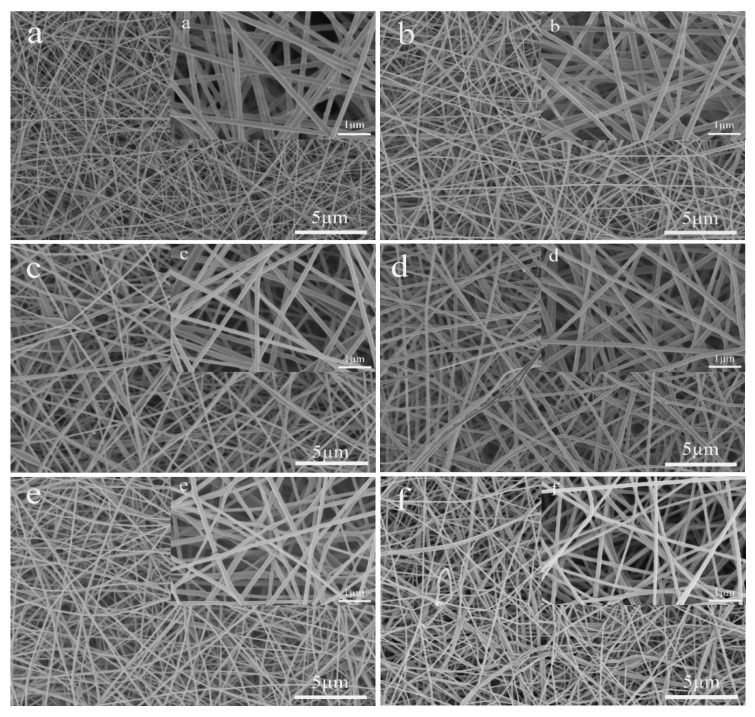
SEM images of PVA nanofibers obtained by different velocity of the air vortex, (**a**) 0 m/s, (**b**) 1 m/s, (**c**) 2 m/s, (**d**) 3 m/s, (**e**) 4 m/s, (**f**) 4.5 m/s. When the velocity of the air vortex is 0 m/s, it is the traditional electrospinning device.

The change of air vortex strength would have a great impact on the morphology of nanofibers. Figure 6 and Table 3 show the SEM images and average diameters of nanofibers prepared via different air vortex strengths. When the velocity of the air vortex is 0 m/s, it is the traditional electrospinning device. It can be seen from Table 3 and Figure 6 that as the velocity of the pumped airflow increases, the average diameter of the nanofibers gradually decreases. This is because when the speed of the pumped airflow increases, the strength of the air vortex formed in the tube will also increase. We know that the vortex has a certain stretching effect on the electrospinning jet. Therefore, when the strength of the air vortex increases, the stretching force of the air vortex on the jet will also increase, and the diameter of the nanofibers will decrease with the increase of the strength of the air vortex. On the other hand, it can be seen from the SEM images that when the incident air velocity is small, that is, when the air vortex intensity is small, the air vortex has little effect on the interaction between the nanofibers. However, when the speed increased to 3 m/s, the air vortex caused the nanofibers to adhere to each other. When the velocity of the pumped air exceeds 3 m/s, as the incident air velocity increases, that is, as the vortex intensity increases, the adhesion between nanofibers becomes more and more serious. This is because when the strength of the air vortex is small, the air vortex only has an effect on the macromolecular chains inside the nanofibers, but when the strength of the air vortex increases to a critical value, it starts to have an effect on the nanofibers, making the nanofibers adhere to each other. And as the strength of the air vortex increases, this force on the nanofibers will also increase. As shown in Figure 6f, when the incident air velocity reaches 4.5 m/s, the adhesion between nanofibers has become very serious. From Table 3, it can be seen that as the intensity of the air vortex increases, the confidence interval was gradually decreasing, which shows that the distribution of nanofibers becomes more and more uniform.

#### 3.2.2. Mechanical Property Test

**Figure 7:**
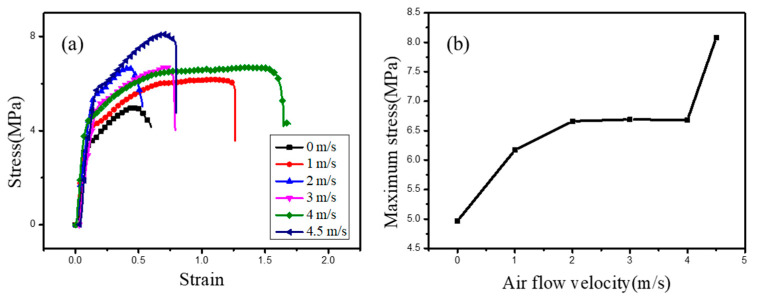
Mechanical property tests for PVA nanofiber membranes prepared in different air vortex strength. (**a**) Mechanical property of nanofiber membrane, (**b**) The maximum stress of nanofiber membranes prepared in different air vortex strength.

From the SEM images we know that when the air vortex strength is weak, it just has effect on macromolecule chains, but once it increases to a critical value, the air vortex begins have effect between nanofibers. So, the air vortex strength will greatly affect nanofiber membranes’ mechanical property. Figure 7 show the mechanical property of nanofiber membranes prepared in different air vortex strength.

It can be seen from Figure 7a that in general the mechanical property of nanofiber membrane increases with the increase of the air vortex strength. This is because when the air vortex strength increased, the entanglement of macromolecule chains become serious, and at the same time nanofibers’ adhesion become serious, too. So, based on the two reasons, the force to overcome the friction among macromolecule chains and nanofibers adhesion will increase, and the mechanical property will be enhanced.

Figure 7b is the maximum stress of nanofiber membranes prepared in different air vortex strength. From this figure we can see that when the velocity of the airflow increased from 0 m/s to 2 m/s, the stress keeps growing trend, but from 2 m/s to 4 m/s, the stress become steady. This maybe because when velocity increased to 3 m/s, the entanglement of macromolecule chains reaches the limitation, and even though the nanofibers begin adhering with each other, but the adhesion was not very serious. Therefore, the stress has little change. However, when the velocity increased to 4.5 m/s, the adhesion become very serious, we can also see this phenomenon from the SEM. So, the force to overcome the friction among nanofibers increased, and the stress increased rapidly.

#### 3.2.3. Gas Permeability

**Figure 8:**
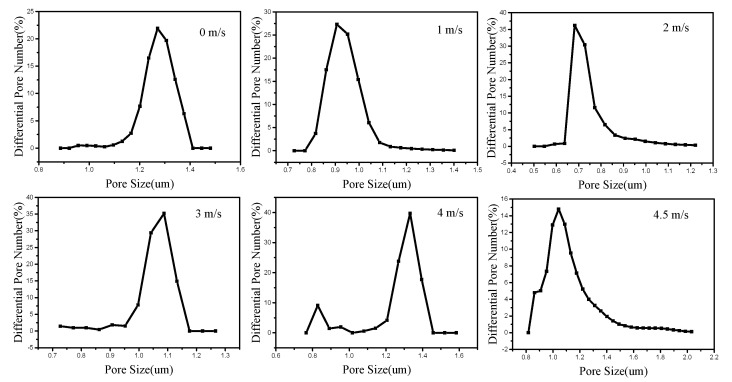
Pore size distributions of PVA membranes prepared in different air vortex strength.

The pore size distributions of PVA membranes obtained in different air vortex strength were measured by a capillary flow porometry. Figure 8 and Table 4 illustrate the pore size distributions of PVA membranes prepared in different air vortex strength.

From Table 4 and Figure 8 we can see that when the air vortex is weak (from 0 m/s to 2 m/s), the pore size decreased first when the air vortex strength increased. This is because the vortex mainly acts on the macromolecular chains inside the nanofibers at this time, and the entanglement of the macromolecular chains becomes more and more compact. The nanofibers are also densely distributed under the action of the vortex, so the pore size of nanofiber membrane gradually decreases. However, when the velocity of the airflow increased to 3 m/s, the pore size begins to gradually increase. This is because the vortex has an effect on the nanofibers and makes the nanofibers adhere to each other. Moreover, as the intensity of the vortex increases, the adhesion between the nanofibers becomes stronger and stronger, so the pore size of the nanofiber membrane will increase. In addition, we can see that the average pore size of nanofiber membrane prepared in the velocity of 4 m/s is similar to the nanofiber membrane prepared in the velocity of 4.5 m/s. This is because the adhesion between nanofibers not happen in every nanofiber, so the adhesion between nanofibers will improve the mechanical property of nanofiber membrane but will not enlarge the average pore size.

## 4. Conclusions

In this paper, for the first time, we use air vortex in the electrospinning process. Compared with traditional electrospinning and cross-certified through the scanning electron microscopy, mechanical property test, Fourier transform infrared spectroscopy and pore size test, we found that weak air vortex in the electrospinning process could control the macromolecule chains’ structure in a nanofiber, making the macromolecule chains entangled with each other and forming a DNA-like structure. As the intensity of the vortex increases, the diameter of the nanofibers gradually decreases. When the vortex strength increases to a certain value, the entanglement of the macromolecular chains inside the nanofiber reaches the limit, and at the same time, the nanofibers begin adhering to each other. As the vortex intensity increases, the mechanical properties of the nanofiber membrane will also increase. Besides, it is also observed that strong air vortex could not only improve the mechanical property of the nanofiber membrane, but also enlarge the pore size, which provide a new and simple method for fabricating high strength and large pore size nanofiber membrane.

In a word, this paper proposes a promising method to control the macromolecule chains structure in a nanofiber and provides an effective and simple method to prepare nanofiber membranes with high strength and large pore size.

## Figures and Tables

**Table 1:** The relationship between the diameter of nanofibers and electrospinning device.

Sample	Average Diameter $\left(\bar{D}\right)$ (nm)	Standard Deviation (σ) (nm)	Confidence Interval (nm)
PVA	178	21.8	±4.3
PVA (vortex)	163	24.5	±4.9
PAN	190	26.4	±7.1
PAN (vortex)	178	26.1	±9.0

**Table 2:** The pore size of PVA and PAN nanofiber membranes.

Sample	Pore Size (μm)	Average Pore Size (μm)	Pore Number
PVA	1.17–1.4	1.27	$3.52\times 10^{8}$
PVA (vortex)	0.817–4.83	0.907	$6.47\times 10^{8}$
PAN	0.817–3.9	0.952	$2.23\times 10^{9}$
PAN (vortex)	0.727–4.83	0.817	$3.5\times 10^{9}$

**Table 3:** The relationship between the air vortex strength and the average diameter of nanofibers.

Airflow Speed (m/s)	Average Diameter $\left(\bar{D}\right)$ (nm)	Standard Deviation (σ) (nm)	Confidence Interval (nm)
0	178	21.8	±4.3
1	163	24.5	±4.9
2	156	23.2	±4.5
3	137	19.6	±3.8
4	130	19.4	±3.8
4.5	107	15.4	±3.0

**Table 4:** The analysis of pore size of PVA membranes prepared in different velocity of air vortex.

Air Vortex Speed (m/s)	Pore Size (μm)	Average Pore Size (μm)	Pore Number
0	1.17–1.4	1.27	$3.52\times 10^{8}$
1	0.817–4.83	0.907	$6.47\times 10^{8}$
2	0.637–4.83	0.817	$6.8\times 10^{8}$
3	0.952–1.17	1.04	$3.54\times 10^{8}$
4	1.14–1.42	1.27	$2.94\times 10^{8}$
4.5	0.952–3.9	1.28	$1.48\times 10^{8}$
